# Hepatitis C Virus Subtypes Novel 6g-Related Subtype and 6w Could Be Indigenous in Southern Taiwan with Characteristic Geographic Distribution

**DOI:** 10.3390/v13071316

**Published:** 2021-07-07

**Authors:** Hung-Da Tung, Pei-Lun Lee, Jyh-Jou Chen, Hsing-Tao Kuo, Ming-Jen Sheu, Chun-Ta Cheng, Tang-Wei Chuang, Hsu-Ju Kao, Yu-Hsun Wu, Mai-Gio Pang, Cheng-Heng Lin, Chia-Yi Hou, Hsin-Hua Tsai, Li-Ching Wu, Chuan Lee

**Affiliations:** 1 Division of Gastroenterology and Hepatology, Department of Internal Medicine, Chi-Mei Medical Center, Liouying, Tainan 73657, Taiwan; d931102@mail.chimei.org.tw (H.-D.T.); peilun57@yahoo.com.tw (P.-L.L.); moon486058@yahoo.com.tw (C.-T.C.); hoffman.i323@msa.hinet.net (T.-W.C.); ju928@hotmail.com (H.-J.K.); B101091076@tmu.edu.tw (Y.-H.W.); pangmaigio@gmail.com (M.-G.P.); skipclown@hotmail.com (C.-H.L.); 2 Division of Gastroenterology and Hepatology, Department of Internal Medicine, Chi-Mei Medical Center, Yongkang, Tainan 71004, Taiwan; kuohsu2003@yahoo.com.tw (H.-T.K.); hmj@mail.chimei.org.tw (M.-J.S.); 3 Department of Clinical Pathology, Chi-Mei Medical Center, Liouying, Tainan 73657, Taiwan; 960360@mail.chimei.org.tw; 4 Department of Clinical Pathology, Chi-Mei Medical Center, Yongkang, Tainan 71004, Taiwan; livelychord@gmail.com (H.-H.T.); 540012@mail.chimei.org.tw (L.-C.W.); 5 Institute of Biomedical Science, National Sun Yat-sen University, Kaohsiung 804201, Taiwan; 6 Division of Gastroenterology and Hepatology, Department of Internal Medicine, Chi-Mei Hospital, Chiali, Tainan 72263, Taiwan; chuanli30@yahoo.com.tw

**Keywords:** hepatitis C virus, genotype, injection drug user, phylogenetic analysis

## Abstract

Hepatitis C virus (HCV) genotype (GT) 6 is the most genetically diverse GT and mainly distributed in Southeast Asia and south China but not Taiwan. Earlier studies showed the major HCV GTs in Taiwan were GT 1b and 2 with very rare GT 6 except in injection drug users (IDUs), and subtype 6a is the main GT 6 subtype among IDUs. Recently, we reported a much higher prevalence (18.3%) of GT 6 in Tainan City, southern Taiwan. This study was designed to clarify the subtypes of GT 6 in this endemic area. A total of 3022 (1343 men and 1679 women) HCV viremic patients were enrolled. Subtypes of GT 6 were determined by sequencing of core/E1 and nonstructural protein 5B in 322 of 518 GT 6 patients. The overall GT 6 prevalence rate was 17.1% (518/3022), with higher prevalence districts (>25%) located in northern Tainan. A novel 6g-related subtype is the most prevalent subtype (81.0%), followed by 6w (10.8%), 6a (7.5%), and 6n (0.7%). The high GT 6 prevalence in Tainan was mainly due to a novel 6g-related subtype and 6w. These two subtypes could be indigenous in Tainan with characteristic geographic distribution.

## 1. Introduction

Determination of hepatitis C virus (HCV) genotypes (GTs) and subtypes is crucial in the understanding of viral evolution, transmission, epidemiology, and treatments selection. Even in the era of interferon-free, pan-genotypic direct acting antivirals (DAAs), some rare subtypes might harbor intrinsic resistance to DAAs [1]. The efficacy of pan-genotypic DDAs might be suboptimal for these local endemic subtypes, as clinical trials covered mainly the global epidemic genotypes/subtypes only [1,2,3,4]. Moreover, the geographic differences in distribution of HCV genotypes and subtypes could reflect the epidemiological history of the virus [5]. Understanding these local endemic subtypes could help to improve public health strategies to prevent further transmission and spreading. HCV GT 6 is the most genetically diverse genotype and is mainly distributed in Southeast Asia, southern China, or immigrants from these area [6,7,8,9,10]. Although Taiwan is an HCV endemic country with an overall prevalence of positive HCV antibody (anti-HCV) of 3–5% in adults, the major prevalent subtypes are 1b and 2a [11,12,13,14,15]. HCV GT 6 infection was seldom reported from Taiwan except among injection drug users (IDUs) and patients with human immunodeficiency virus (HIV) infection, and the major GT 6 subtypes were 6a, 6k, and 6n [16,17]. We recently reported an unexpected high prevalence (18.3%) of HCV GT 6 in Tainan City, with remarkably geographic restriction between two rivers. To clarify the epidemiology of this unique HCV GT in southern Taiwan as a local endemic disease and geographic variation of GT 6 subtypes, we further determined the subtypes of GT 6 and analyzed their distribution in Tainan City.

## 2. Materials and Methods

### 2.1. Study Population

**Figure 1:**
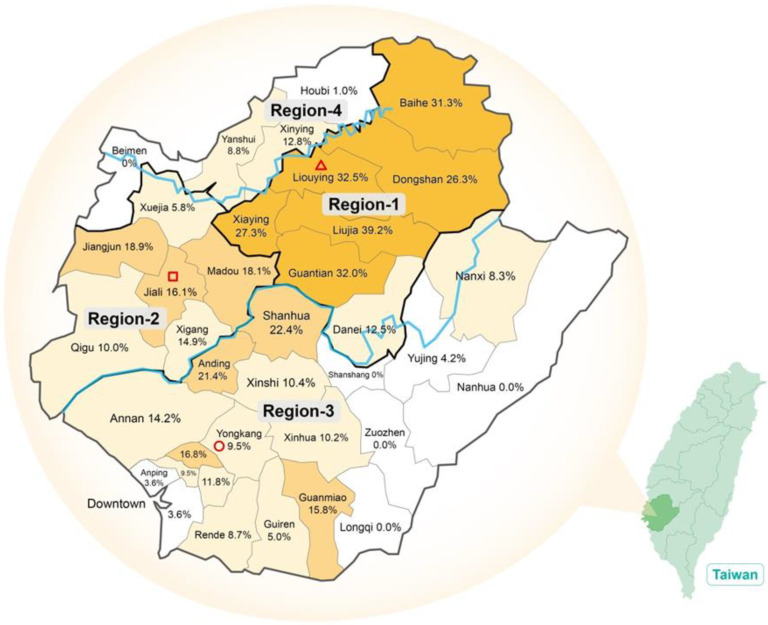
Prevalence rates of HCV GT 6 in each district of Tainan. (Blue lines: two main rivers, top: Jishui; middle: Zengwen; red hollow circle, triangle and square: locations of three campuses).

**Figure 2:**
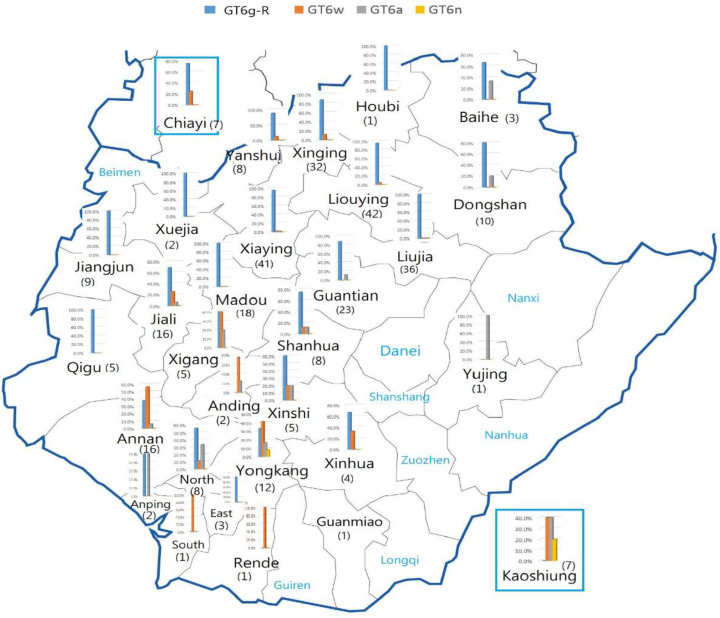
Percentages and numbers of major HCV GT 6 subtypes in Tainan.

From 1 March 2016 to 28 February 2019, a total of 3339 patients with HCV viremia who were followed up at Chi Mei medical system, including Yongkang, Liouying, and Chiali (Jiali, in Figure 1 and Figure 2) campuses for reimbursement of DAAs treatment were enrolled, including 317 patients who are not residents of Tainan city (Kaohsiung City and Chiayi City/County, at south and north of Tainan). There were 3022 (1343 men and 1679 women, mean age, 64.6 ± 12.4 years) patients who live in 37 districts of Tainan City. We excluded the Lonqgi district, for only one patient was registered in our database. The definition of patients living district was according to patients’ chart records.

### 2.2. Diagnostic Procedures

#### Study Design

The working flow of HCV genotype determination had been reported previously [18,19]. In brief, the Abbott Realti*m*e Genotype II (Des Plaines, IL, USA) was used to determine HCV GTs successfully in 2568 patients, then Abbott Realti*m*e Genotype II PLUS assay (Des Plaines, IL, USA) was used when Realti*m*e GT II revealed ambiguous results (GT 1 without subtype, indeterminate, mixed, or undetected) in 567 patients. Nonstructural protein 5B (NS5B) and core/E1 sequencing were conducted to check genotypes when PLUS assays still could not determine the genotypes in 204 patients.

### 2.3. Genotyping by Abbott GT II and PLUS Assays

For analysis GTs with the Abbott GT II and *PLUS* assays, RNA was extracted from the 300 μL serum on the Abbott *m*2000sp system using the Abbott *m*Sample Preparation System kit (Des Plaines, IL, USA) according to the manufacturer’s instruction. Reverse transcription (RT)-PCR master mixes were set up using the Abbott *m*2000sp and Abbott RealTi*m*e HCV Genotype II Amplification Reagent Kit or the RealTi*m*e HCV Genotype *PLUS* Amplification Reagent Kit (Des Plaines, IL, USA). RT-PCR reactions were performed using the Abbott m2000rt instrument.

### 2.4. Genotyping by 5′UTR/Core Sequencing

HCV RNA was extracted from 300 μL patient’s serum; nested PCRs were performed using Invitrogen kit to amplify the 5′UTR and core regions. For cDNA synthesis and the nested PCR, in the first PCR round, the outer primers corresponding to HCV-1 H77 sequence -268 to -251 (5′-AGCGTCTAGCCATTGGCGT-3′) and antisense primer core 391–410 (5′-ATGTACCCCATGAGGTCGGC-3′) were used [20]. Nested PCR primers were -199 to -183 (5′-GTGGTCTGCGGAACCGG-3′) and antisense core 364–383 (5′-CAC/TGTA/GAGGGTATCGATGAC-3′) [21,22]. PCR products lengths were 681 and 589 bps, respectively, which were purified and sequenced for analysis. Sequences generated were aligned using Basic Local Alignment Search Tool (BLAST) of NCBI database to determine genotypes.

### 2.5. Subtyping by E1 and NS5B Sequencing

HCV RNA was extracted from 300 μL of serum using LabTurbo viral Mini Kit (Taigen, Taipei, Taiwan) according to the recommended protocol. cDNA and PCR was synthesized from 10 μL extracted RNA with Superscript™ One-Step RT-PCR system (Invitrogen, Life Technologies, Carlsbad, CA, USA) and outer primers specific for the E1 and NS5B. Sequencing of the E1 and NS5B regions was achieved by nested PCR using 1~2 μL PCR product, inner primers, and GoTaq^®^ Master Mixes (Promega, Madison, WI, USA). The primers of the partial E1 region (reference strain H77 positions: 847–1325 nt) for nested PCR were described below: outer forward (E1) 5′-GGATYAAYTATGCAACAGGGAATCTWCCYGG-3′, outer reverse (E2) 5′-GTCCKRTTTATRTGCCARCTGCCRTT-3′; inner forward (E3) 5′-TTCGCCGACCTCATGGGGTACAT-3′, inner reverse (E4) 5′-GGACCAGTTCATCATCATATCCCA-3′. The primers of the partial NS5B region (reference strain H77 positions: 8297–8690 nt) for nested PCR are described below: outer forward (N1) 5′-GTGGCGCTCMAAGAARACYCCWATGGG-3′, outer reverse (N2) 5′-CCAGGARTTSACTGGAGTGTGNCGRGCBGT-3′; inner forward (N3) 5′-TGGGNTTYTCTTAYGACACCAGRTGYTTTGA-3′, inner reverse (N4) 5′-TACCTGGTCATAGCNTCCGTGAANGCTC-3′. One-Step RT-PCR using outer primers was conducted with the following conditions: 48 °C for 40 min of cDNA synthesis and PCR stage by 95 °C for 5 min, 35 cycles of 95 °C for 1 min, 55 °C for 1min, and 72 °C for 2 min, then a final cycle at 72 °C for 10 min. Second round PCR using inner primers was conducted with the following conditions: 95 °C for 5 min, 35 cycles of 95 °C for 30 s, 55 °C for 30 s, and 72 °C for 1 min, then a final cycle at 72 °C for 10 min. The amplicons were identified on 2.0% agarose gel. To avoid potential contamination, experimental procedures were strictly performed by adding negative controls and processed in parallel, including preparation of reagents, RT-PCR, nested PCR, and gel electrophoresis.

### 2.6. Phylogenetic Analysis by E1 and NS5B Sequencing

Sequencing was done with primers E3 and N3 using ABI Prism Big Dye 3.0 terminators on an ABI 3730xl DNA Analyzer (PE Applied Biosystems, Foster City, CA, USA). The nucleotide sequences obtained were aligned with HCV strains of standard genotypes and edited by the ClustalW method of MEGA software (Version X) [23]. The phylogenetic analysis was inferred using the neighbor-joining method. The percentages of replicate trees in which the associated taxa clustered together in the bootstrap test (1000 replicates) are shown next to the branches. The tree is drawn to scale, with branch lengths in the same units as those of the evolutionary distances used to infer the phylogenetic tree. The evolutionary distances were computed using the Kimura 2-parameter method and are in the units of the number of base substitutions per site. Codon positions included were 1st+2nd+3rd+Noncoding. All positions with less than 95% site coverage were eliminated, i.e., fewer than 5% alignment gaps, missing data, and ambiguous bases were allowed at any position (partial deletion option).

The study was approved by ethical committee of Chi Mei Medical Center, and informed consent was obtained from each patient. All experiments were performed in accordance with relevant guidelines and regulations. The study was conducted according to the guidelines of the Declaration of Helsinki.

The significance of possible associations between discrete variables was compared using chi-square test. The continuous variables were compared with student t test. The level of statistical significance was set at two-tailed *p* < 0.05.

## 3. Results

The overall numbers and prevalence rates of HCV GT 1a, 1b, 2, 3, 4, 6, and mixed types among chronic hepatitis C (CHC) patients were 119 (3.9%), 956 (31.6%), 1388 (45.9%), 19(0.6%), 7 (0.2%), 518 (17.1%), and 15 (0.5%), respectively (Table 1). There were no GT 5 and very rare GT 3 and 4 (combined less than 1%). Genotypes 2 and 1b remained two major prevalent HCV genotypes in Tainan, as supported by previous studies, but with higher GT 2 and much lower GT 1b prevalence [11,12,13,14]. HCV GT 6 prevalence rate 17.1% was significantly higher than previous studies reported but similar to our previous, smaller study (18.3%) [18]. Numbers and percentages of each HCV GT of 36 districts are shown in Table 1 and Figure 1.

Two major rivers, Jishui River (JR) and Zengwen River (ZR), run through the Tainan city from eastern mountainous area to the western coast (blue lines in Figure 1). We divided into four regions according to geographic and HCV prevalence variations (thick black lines in Figure 1). From east to central, the upper stream area between JR and ZR was assigned as Region-1, including seven districts; the downstream area between JR and ZR was assigned as Region-2 with six districts; the southern area of ZR, almost half of Tainan city with 19 districts, we assigned as Region-3; and the northern area of JR was assigned as Region-4 with only four districts. Eleven of 13 districts with high HCV GT 6 prevalence > 15% showed cluster between or adjacent to these two major rivers. All districts except one (Xuejia) located between JR and ZR (region 1 and 2) have prevalence of GT 6 more than 10% (Figure 1).

Region-1 has the highest prevalence of HCV GT 6, 31.4% (266/847); six of seven (85.7%) districts showed prevalence of GT 6 more than 25% (Figure 1), especially Liujia, with the highest (39.2%) GT 6 prevalence; even the lowest-prevalence district (Danei) has 12.5%. Genotype 1a, 1b, 2, 3, and 4 comprise 1.9, 26.9, 38.3, 0.6, and 0.1%, respectively (Figure 1, Table 2).

Region-2 has the highest GT 2 prevalence (51.7%). The prevalence rates of GT 1a, 1b, 2, 4, and 6 were 4.1, 28.2, 51.7, 0.4, and 15.3%, respectively. In fact, one coastal village (Masago of Jiangjun District) was reported to have the highest HCV GT 2 prevalence (63.5%) so far ever reported in Taiwan [24]. All these districts except one (Xeujia 5.8%) have prevalence of GT 6 > 10%.

Region-3 consists of 19 districts of southern Tainan. The prevalence rates of HCV GT 1a, 1b, 2, 3, 4, and 6 are 4.8, 31.8, 50.8, 1.2, 0.4, and 10.7%, respectively (Table 2). Two districts with higher GT 6 prevalence of 22.4% and 21.4%, Shanhua and Anding, are located close to southern border of ZR. The prevalence rates of the rest districts are all under 15% except for two districts (North and Guanmiao districts, 16.8 and 15.8%, respectively).

Region-4 consists of Houbi, Xinying, Yanshui, and Beimen, the four districts of northern Tainan. The prevalence rates of HCV GT 1a, 1b, 2, 3, and 6 are 5.3, 41.8, 42.7, 0.2, and 9.6%, respectively. Xinying, with the highest GT 6 prevalence of 12.8%, is located close to one high-GT 6 district in Region-1, Liouying (32.5%).

The HCV GTs distribution of Region-1 showed significantly higher prevalence of GT 6 (Region-1 vs. Region-2, 3, 4; *p* < 0.00001) and lower prevalence of GT 1b (Region-1 vs. Region-3, 4; *p* < 0.05) and GT 2 (Region-1 vs. Region-2, 3; *p* < 0.00001).

The subtyping results were successfully obtained in 322 of 331 (97.3%) of core/E1 and 320 of 331 (96.7%) of NS5B sequences of CHC patients with GT 6 (including seven patients from Chiayi and seven patients from Kaohsiung, at the north and south of Tainan city, respectively) with available sera for PCR and sequencing. Subtyping results were concordant in 307 of 331 (92.7%) of samples. The circular phylogenetic tree generated using E1 sequences with prototypes of GT 1~5, 7, and 8 and GT 6 subtypes confirmed these samples classified as GT 6 (Supplement Figure S1). However, most of our samples were not subtype 6g, as previously assumed (Supplemental Figure S2) [18,19]. Instead, these samples clustered with a novel subtype (closely related to 6g) reported by Hedskog et al. [25]. This patient was enrolled for Sofosbuvir-based therapy from the Liouying campus, and her HCV was typed as GT 1b [25]. It is known that “confirmation of a new subtype requires a complete or nearly complete coding region sequence differing from other sequences by at least 15% of nucleotide positions and sequence information from at least two other isolates in core/E1 (>90% of the sequence corresponding to positions 869 to 1292 of the H77 reference sequence accession number AF009606) and NS5B (>90% of positions 8,276 to 8,615)” [7], and the sequences of our samples covered 90.6% of core/E1 (909 to 1309) and 88.8% of NS5B (8316 to 8624) that are required for confirmation of new subtypes. Hence, we can almost ascertain that a new subtype closely related to 6g is identified and confirmed (6xj). Even in the most distantly related sample (10784309), the homology of core/E1 and NS5B sequences between our samples and this novel subtype are still greater than 85% (85.2 and 92.2%, respectively, data not shown). Meanwhile, there are three samples (10514010; 21004325; and 10134166) clustered together that could be another potential novel subtype (closely related to 6w) once a complete coding region sequence is available (Supplement Figure S3A,B).

Subtypes of HCV GT 6 that we determined in this area included 6a, g-related, n, t, v, and w, but only four subtypes—6a, g-related, n, and w—showed concordant typing results between core/E1 and NS5B regions. This novel 6g-related subtype is the most prevalent subtype in 79.8% (245/307) of HCV GT 6 samples (81.0%, 239/295 of Tainan samples), followed by 6w 11.4% (35), 6a 7.5% (24), and 6n 1.0% (3) (Table 3, Figure 2).

The geographic distribution and ratios of 307 HCV GT 6 subtypes from 29 districts of Tainan, Chiayi, and Kaohsiung are summarized in Table 3 and Figure 2. The distribution of 6w is clearly located in southwestern Tainan, mainly south of ZR, while the novel 6g-related subtype is distributed at north of Tainan, especially in Region-1, and Chiayi but not Kaohsiung. Subtype 6a was found in downtown area and mountainous area of upstream of ZR. HCV GT 6 infection is relatively rare in hilly southeastern Tainan.

**Figure 3:**
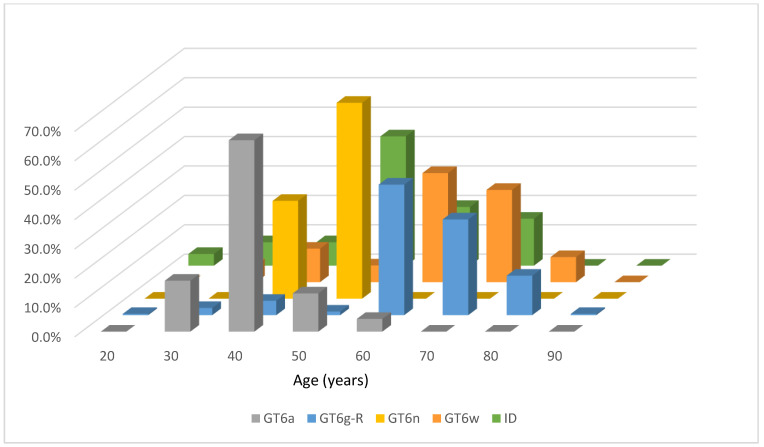
Percentages of age distribution of HCV GT 6 subtypes. ID, indeterminate.

The age distribution of HCV GT 6 subtypes is depicted in Figure 3. Subtypes 6g-related and 6w were significantly older than 6a and 6n, suggesting different time and routes of transmission existed in these patients.

## 4. Discussions

In this study, more than five hundred HCV viremic patients carried GT 6 in Tainan, southern Taiwan. This is the largest number of HCV GT 6 infections ever reported in Taiwan. In contrast to the peninsular Southeast Asia, with endemic HCV GT 6 infection, GT 6 infection is rarely reported in community or hospital-based studies from Taiwan. In an earlier study from southern Taiwan, only 2 of 418 (0.5%) HCV patients were infected with GT 6a [13]. One of the reasons of such low HCV GT 6 prevalence in earlier studies could be the limitation of earlier genotyping assays. Earlier studies using line-probe assay or PCR with type-specific primers aimed for 5′ untranslated region (5′UTR) for genotypes determination could not detect GT 6 [11,12,26,27]. High genetic conservation of 5′UTR is suitable for PCR amplification and RNA detection but lacks sufficient variation to distinguish some GT 1 and 6 and some subtypes [28,29,30]. We have found a high rate of HCV GT 1 without subtype designation using Abbott Realti*m*e HCV GT II assay for HCV genotyping in our medical system since 2016. Among these GT 1 without subtype designation, nearly 80% were confirmed to be GT 6 by Abbott Realti*m*e HCV GT II PLUS and core/E1 sequencing [18]. Besides subtypes 6a and 6b, Abbott Realti*m*e HCV GT II PLUS assay detects more GT 6 subtypes (6c–6l) and subtypes 1a and 1b in a single reaction [31]. In our previous study, 163 of 210 samples with ambiguous result by Abbott Realti*m*e HCV GT II were identified as GT 6 by GT II PLUS [18]. Hence, it is reasonable to assume that such a higher GT 6 detection rate was due to increased detection of novel 6g-related subtype in this study.

Another reason of low HCV GT 6 prevalence in earlier reports from Taiwan might be that the distribution of HCV GT 6 is highly geographic restricted in Tainan, as this novel subtype was mainly restricted between two main rivers, and 6w was restricted in southern Tainan, suggesting a local endemic disease with considerable duration. Also, 6g-related subtype was found in Chiayi (north of Tainan) but not Kaohsiung (south of Tainan), albeit the case numbers are small.

Four HCV GT 6 subtypes, including 6g-related, w, a, and n, were identified in this study, but only this novel 6g-related subtype was first reported in Taiwan (misjudged as 6g simply from BLAST results) [18,19]. HCV GT 6, 3, and 1a were highly prevalent among IDUs, with HIV and subtype 6a having been reported as the main GT 6 subtype (23.5~37.9%) [16,17,32], followed with 6 g, k, n, and w. extremely high anti-HCV prevalence rates from 96.6~98.7% among IDUs with HIV had been reported in Taiwan [17,32,33]. This is mostly because an outbreak of HIV with recombinant circulating form (CRF) 07_BC was spread among IDUs in Taiwan since 2003 and peaked in 2005 [34,35] along with even greater HCV outbreak since both viruses share similar transmission routes and even higher transmission efficiency [16]. A molecular epidemiological study showed these CRF07_BC HIV sequences from Taiwan resemble the dominant strains circulating among IDUs in China [35]. Evolutionary analyses also revealed that CRF07_BC was introduced into southern Taiwan in 1998–2001 and spread to central-northern Taiwan in 2001–2003 [36], causing the largest HIV/AIDS and also HCV outbreaks in Taiwan [16,17,32,34,35,37]. The origins of CRF07_BC HIV and HCV could be traced to Yunnan Province, China, and transmitted via heroin-trafficking routes [17,38]. Interestingly, 6g and 6w had not been reported in Yunnan and peninsular Southeast Asia but from Hong Kong, Guangzhou, China, and Indonesia [39,40,41]. In a large study of HCV genotypes and subtypes circulating in China, 4 GTs and 18 subtypes were identified among 32,030 patients; hepatitis C virus GT 6 infections were detected in 2332 samples (7.28%), with the most prevalent being 6a (2045/2332), followed by 6n (226), 6u (36), 6g (4), 6v (2), 6w (2), and 6e, b, j, q, and r (each 1) [42]. Given the large number of old-aged, 6g-related and 6w and less than 10% younger 6a/6n patients in our study, it seemed less likely that 6g-related and 6w were transmitted from China to Taiwan or disseminated from IDUs after 2003 outbreak. Subtype 6a and 6n were transmitted from China and Southeast Asia via heroin-trafficking routes, as these patients who carried subtype 6a and 6n were significantly younger and with higher ratio of self-reported IDU history (also GT 1a, data not shown). From the phylogenetic trees of E1 and NS5B, 6w D140, and D370 prototypic stains clustered closely with our samples (Supplement Figure S3A,B), further supporting that 6w detected among IDUs could be spilled over from this area, as only two 6w and one 2b6w recombinant had been reported among hundreds IDUs/HIV in Taiwan [16,17,32]. The ages, transmission history and epidemiology of these patients with novel 6g-related subtype and 6w were obviously different from GT 6a/n patients.

Studies from Hainan island of China, which is close to Vietnam, reported a unique ecosystem of Li tribe (controversially as Austronesian descendants) with HCV infection maintained over 600 years with many novel GT 6 strains sharing a common ancestor with subtype 6g and 6w dating from the end of the 12th century [43,44,45]. From the evolutional and time-scaled phylogenetical analyses, HCV subtypes 6g and 6w have co-evolved and diverted from group of GT 6 subtypes c~f, o~u, xc, xf, and xh for centuries [6,7,9,46,47]. Similarly, there are three samples clustered but not belonging to 6w or 6g-related subtypes that comprise a potential novel subtype. These three samples shared a common ancestor with 6g, w, and 6g-related subtypes, suggesting a long-term HCV infection history existed in Tainan as Hainan island studies shown. Interestingly, the divergence of HCV in Tainan was less prominent than Hainan island. About 80% of GT 6 infections were due to this novel 6g-related subtype. Such a closely related novel 6g-related subtype distribution could be due to the founder effect of the small population of indigenous people who resided in the ancient Taiwan island. Before Hans people immigrated to Taiwan in the 17th century, the population size of indigenous people might have been able to sustain low levels of HCV transmission and infection. Hence, only a few HCV strains were maintained and circulated in this area until more efficient transmission routes were introduced and caused endemic spread in the 20th century. This scenario was similar to the GT 6 history observed in Southeast Asia [6]. The surging of HCV-related HCC in southwest Taiwan after 1980 also supports this observation [48].

In contrast to the Hainan Li tribe population that resided in a relatively closed environment, Taiwan has been populated by Austronesian populations for six-thousand years, occupied by Ming-Qing dynasties for hundreds of years, and colonized by Spain, Netherland, and Japan for decades, also with massive populations migrating from China to Taiwan after World War II. The epidemiological history of HCV among the indigenous Austronesian tribe (Siraya tribe, plains indigenous people) who resided in Tainan for centuries seemed more difficult to recover.

In conclusion, from the geographic restriction, chronological events, and evolutional and phylogenetic evidences, we identified that novel 6g-related and 6w HCV subtypes could be indigenous in Tainan, southern Taiwan. The infection history might last several hundred years from the phylogenetic evidence with new subtypes evolution. Subtype 6w among IDUS could be spilled over from this area. This should be able to prevent stigmatization of these older HCV GT 6 patients as IDU related and also shed some light on the understanding of HCV GT 6 history in Southeast Asia. New emerging strains such as 6a, k, and n as well as 1a and 3 were most likely spread along HIV CRF07_BC outbreak after the 21st century. Existence of potentially new subtypes could be anticipated, and the phylogenetic relatedness with Hainan strains is awaiting for further complete genome data to answer.

## Figures and Tables

**Table 1:** Distribution of HCV genotypes of 36 districts of Tainan city.

Region	Districts	GT1a	GT1b	GT2	GT3	GT4	GT6	MT	T (n)
1	Baihe	0	6	5	0	0	5	0	16
	Dongshan	1	21	36	0	0	21	1	80
	Liouying	4	53	75	0	0	65	1	198
	Liujia	2	30	70	1	0	67	1	171
	Xiaying	3	82	83	2	0	65	3	238
	Guantian	5	31	48	1	1	41	1	128
	Danei	1	5	7	1	0	2	0	16
2	Xuejia	2	27	20	0	0	3	0	52
	Madou	12	25	66	0	1	23	1	128
	Jiangjun	2	19	82	0	0	24	1	128
	Chiali	6	45	63	0	1	22	0	137
	Xigang	1	17	22	0	0	7	0	47
	Qigu	0	26	38	0	0	7	0	71
3	Nanxi	0	5	6	0	0	1	0	12
	Yujing	1	9	13	0	0	1	0	24
	Nanhua	1	3	3	0	0	0	0	7
	Shanshang	1	1	5	0	0	0	0	7
	Zuozhen	0	0	2	0	0	0	0	2
	Shanhua	6	12	20	0	0	11	0	49
	Xinshi	2	16	23	1	0	5	1	48
	Xinhua	0	28	49	0	1	9	1	88
	Guanmiao	1	5	10	0	0	3	0	19
	Anding	0	4	7	0	0	3	0	14
	Yongkang	10	75	116	7	0	22	1	231
	Guiren	3	11	24	0	0	2	0	40
	Annan	8	42	69	1	1	20	0	141
	Anping	2	27	51	0	1	3	0	84
	North	6	35	36	1	0	16	0	94
	East	2	23	33	2	0	8	0	68
	W. central	1	16	21	0	0	4	0	42
	South	3	13	35	1	1	2	0	55
	Rende	3	8	9	0	0	2	1	23
4	Houbi	16	44	39	0	0	1	0	100
	Xinying	12	146	132	1	0	43	1	335
	Yanshui	2	44	57	0	0	10	1	114
	Beimen	0	2	13	0	0	0	0	15
	Total	119	956	1388	19	7	518	15	3022

**Table 2:** Geographic variation of prevalence of different HCV genotypes in Tainan.

	Region-1		Region-2		Region-3		Region-4		Total	
	*n*	%	*n*	%	*n*	%	*n*	%	*n*	%
GT 1a	16	1.9%	23	4.1%	50	4.8%	30	5.3%	119	3.9%
GT 1b	228	26.9%	159	28.2%	333	31.8%	236	41.8%	956	31.6%
GT 2	324	38.3%	291	51.7%	532	50.8%	241	42.7%	1388	45.9%
GT 3	5	0.6%	0		13	1.2%	1	0.2%	19	0.6%
GT 4	1	0.1%	2	0.4%	4	0.4%	0		7	0.2%
GT 6	266	31.4%	86	15.3%	112	10.7%	54	9.6%	518	17.1%
Mixed GT	7	0.8%	2	0.4%	4	0.4%	2	0.4%	15	0.5%
Total	847	28.0%	563	18.6%	1048	34.7%	564	18.7%	3022	

**Table 3:** Distribution of subtypes of 331 HCV GT 6 patients of 29 districts of Tainan city, Chiayi, and Kaohsiung.

Region	Districts	GT6a	%	GT6g	%	GT6n	%	GT6w	%	ID	%	Total
1	Baihe	1	33.3%	2	66.7%	0	0.0%	0	0.0%	0	0.0%	3
	Dongshan	1	10.0%	4	40.0%	0	0.0%	0	0.0%	5	50.0%	10
	Liouying	0	0.0%	38	90.5%	0	0.0%	2	4.8%	2	4.8%	42
	Liujia	0	0.0%	34	94.4%	0	0.0%	0	0.0%	2	5.6%	36
	Xiaying	1	2.4%	39	95.1%	0	0.0%	1	2.4%	0	0.0%	41
	Guantian	3	13.0%	19	82.6%	0	0.0%	0	0.0%	1	4.3%	23
		6	3.9%	136	87.7%	0	0.0%	3	1.9%	10	6.5%	155
2	Xuejia	0	0.0%	2	100.0%	0	0.0%	0	0.0%	0	0.0%	2
	Madou	0	0.0%	18	100.0%	0	0.0%	0	0.0%	0	0.0%	18
	Jiangjun	0	0.0%	9	100.0%	0	0.0%	0	0.0%	0	0.0%	9
	Jiali	0	0.0%	11	68.8%	1	6.3%	4	25.0%	0	0.0%	16
	Xigang	1	20.0%	2	40.0%	0	0.0%	2	40.0%	0	0.0%	5
	Qigu	0	0.0%	4	80.0%	0	0.0%	0	0.0%	1	20.0%	5
		1	1.8%	46	83.6%	1	1.8%	6	10.9%	1	1.8%	55
3	Yujing	1	100.0%	0	0.0%	0	0.0%	0	0.0%	0	0.0%	1
	Shanhua	1	12.5%	6	75.0%	0	0.0%	1	12.5%	0	0.0%	8
	Xinhua	0	0.0%	2	50.0%	0	0.0%	1	25.0%	1	25.0%	4
	Xinshi	1	20.0%	3	60.0%	0	0.0%	1	20.0%	0	0.0%	5
	Guanmiao	1	100.0%	0	0.0%	0	0.0%	0	0.0%	0	0.0%	1
	Yongkang	2	16.7%	3	25.0%	1	8.3%	4	33.3%	2	16.7%	12
	Anding	1	50.0%	0	0.0%	0	0.0%	1	50.0%	0	0.0%	2
	Annan	1	6.3%	5	31.3%	0	0.0%	9	56.3%	1	6.3%	16
	Anping	1	50.0%	1	50.0%	0	0.0%	0	0.0%	0	0.0%	2
	North	2	25.0%	5	62.5%	0	0.0%	1	12.5%	0	0.0%	8
	East	0	0.0%	3	100.0%	0	0.0%	0	0.0%	0	0.0%	3
	W. central	0	0.0%	0	0.0%	0	0.0%	0	0.0%	2	100.0%	2
	South	0	0.0%	0	0.0%	0	0.0%	1	100.0%	0	0.0%	1
	Rende	0	0.0%	0	0.0%	0	0.0%	1	100.0%	0	0.0%	1
		11	16.7%	28	42.4%	1	1.5%	20	30.3%	6	9.1%	66
4	Houbi	0	0.0%	1	100.0%	0	0.0%	0	0.0%	0	0.0%	1
	Xinying	4	12.5%	22	68.8%	0	0.0%	2	6.3%	4	12.5%	32
	Yanshui	0	0.0%	6	75.0%	0	0.0%	1	12.5%	1	12.5%	8
		4	9.8%	29	70.7%	0	0.0%	3	7.3%	5	12.2%	41
	Chiayi	0	0.0%	6	85.7%	0	0.0%	1	14.3%	0	0.0%	7
	Kaohsiung	2	28.6%	0	0.0%	1	14.3%	2	28.6%	2	28.6%	7

Excluded 6 districts: Danei, Nanxi, Nanhua, Zuozhen, Shanshang, and Guiren because no sample available for subtype analysis.

## Data Availability

The data that support the findings of this study are available from the corresponding author upon reasonable request.

## Supplementary Materials

The following are available online at https://www.mdpi.com/article/10.3390/v13071316/s1, Figure S1: Circular neighbor-joining phylogenetic tree of E1 sequences of GT 6 samples with confirmed GT 6 subtypes and GT 1-5, 7 and 8; Figure S2: Radiation neighbor-joining tree of E1 sequences of GT 6 samples with confirmed GT 6 subtypes and GT 1-5, 7 and 8; Figure S3: Neighbor-joining trees of ten 6g-related, ten 6w, three 6n and 3 potential new subtype sequences with confirmed GT 6 subtypes and 6g-related novel subtypes from Hainan island, A: E1, B: NS5B.
