# Supplementary figures and images for: Hepatitis C Virus Subtypes Novel 6g-Related Subtype and 6w Could Be Indigenous in Southern Taiwan with Characteristic Geographic Distribution
Source: Viruses. 2021 Jul 7;13(7):1316. doi: 10.3390/v13071316 (PMC8310057; doi:10.3390/v13071316)

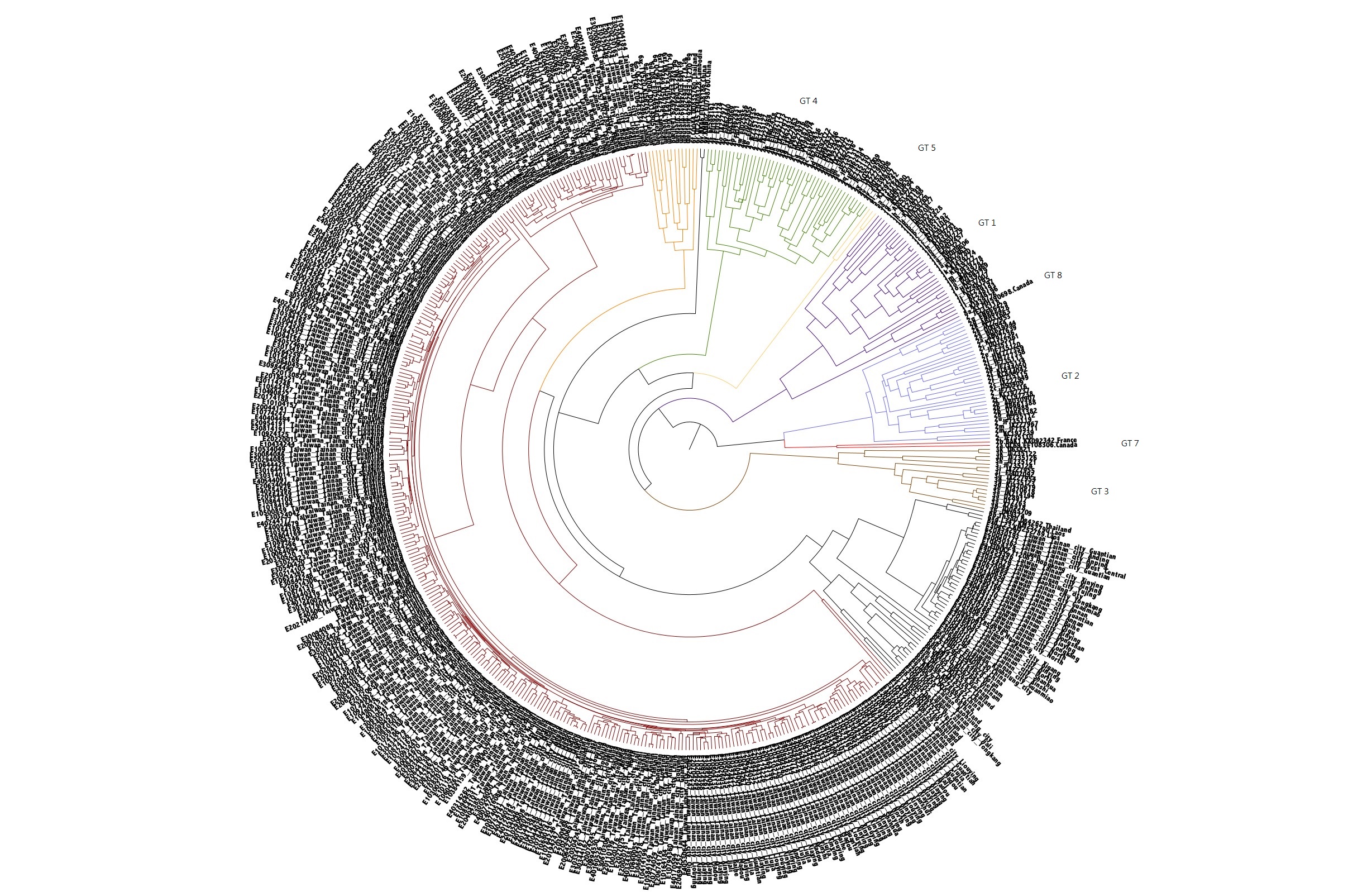

Supplement: Supplementary file 1 [file viruses-13-01316-s001.zip › Figure S1. E1 sequences with GT 1-8 circular.jpg]

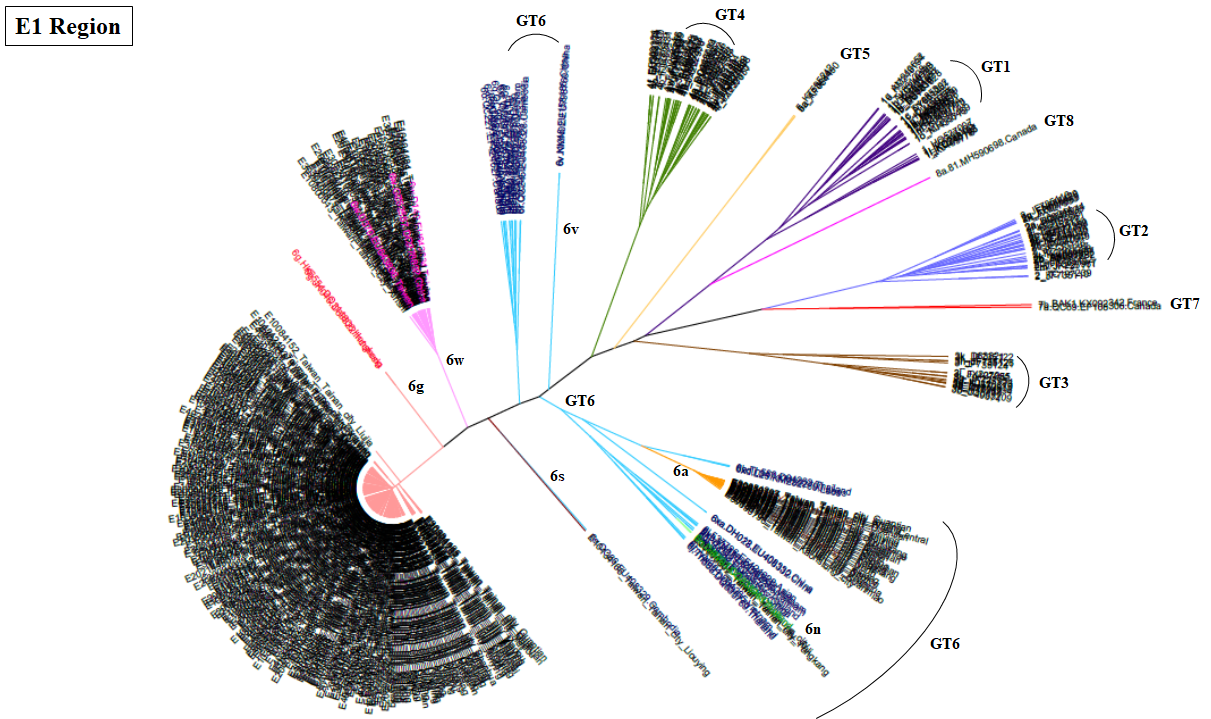

Supplement: Supplementary file 1 [file viruses-13-01316-s001.zip › Figure S2. 6g related new subtype radiation.png]

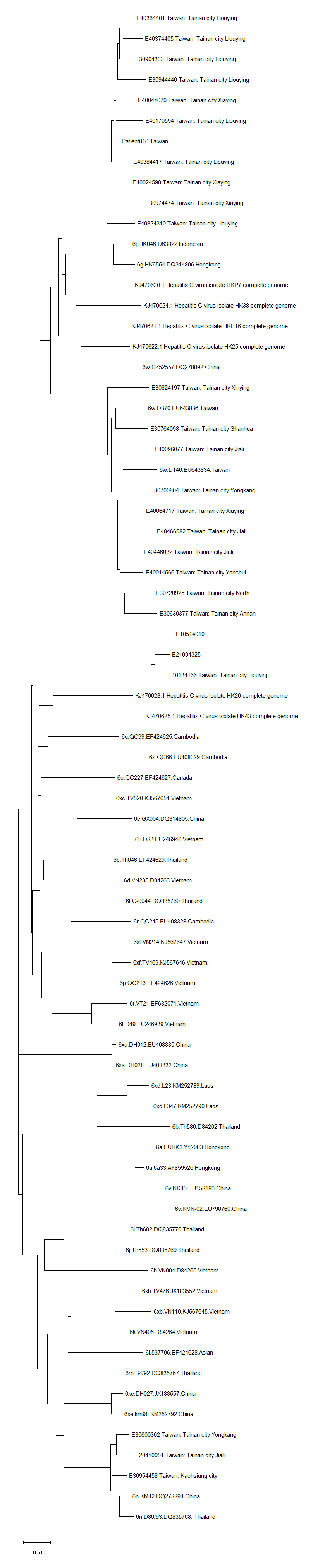

Supplement: Supplementary file 1 [file viruses-13-01316-s001.zip › Figure S3A. E1 sequences with GT 6 subtypes.png]

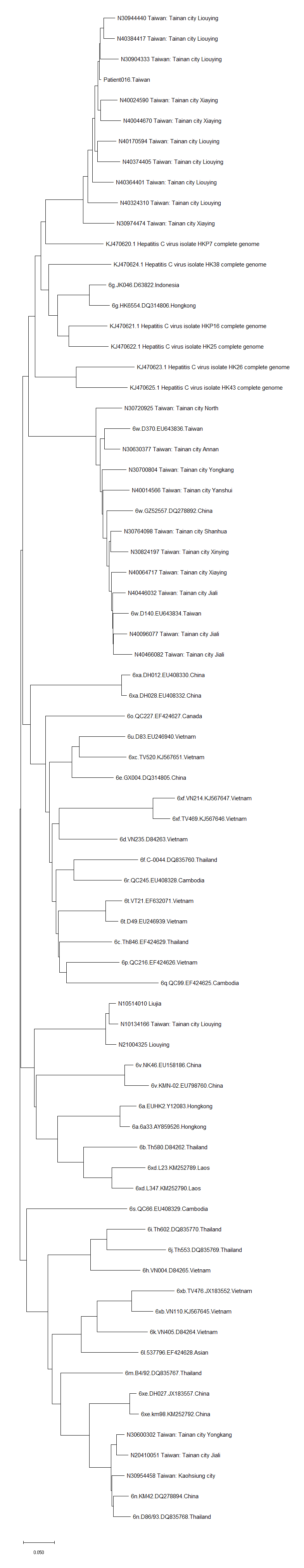

Supplement: Supplementary file 1 [file viruses-13-01316-s001.zip › Figure S3B. 5B sequences with GT 6 subtypes.png]
